# Post-operative practice patterns after the bidirectional Glenn surgery: a survey

**DOI:** 10.1017/S104795112511113X

**Published:** 2026-01-23

**Authors:** Bennett Weinerman, Soon Bin Kwon, Eva W. Cheung, Soojin Park

**Affiliations:** 1 Division of Pediatric Critical Care and Hospital Medicine, Columbia University College of Physicians and Surgeons, New York, NY, USA; 2 Program in Hospital and Intensive Care Informatics, Department of Neurology, Columbia University Vagelos College of Physicians and Surgeons, New York, NY, USA; 3 Department of Neurology, Columbia University Vagelos College of Physicians and Surgeons, New York, NY, USA; 4 NewYork-Presbyterian Hospital, Columbia University Irving Medical Center, New York, NY, USA; 5 Department of Biomedical Informatics, Columbia University, New York, NY, USA

**Keywords:** Paediatric, congenital heart disease, bidirectional Glenn, paediatric cardiac intensive care

## Abstract

**Objectives:**

The bidirectional Glenn surgery is an important staging procedure for patients with single ventricle physiology. Approximately 1000 children are born each year in the United States with this subset of CHD. There is limited data regarding optimal post-operative management for these children. We surveyed paediatric cardiac intensive care providers surrounding their management strategies after the bidirectional Glenn surgery.

**Design:**

An anonymous survey was distributed via email to paediatric cardiac intensive care providers. The survey included anonymised demographic data and focused on post-operative physiologic targets for patients recovering after the bidirectional Glenn surgery.

**Subjects:**

Thirty-five paediatric cardiac intensive care providers responded to an anonymous 12-question survey. Subjects were mostly comprised of paediatric cardiac intensive care attendings (80%), with an average of 7.86 years of training. The respondents primarily practised in settings with medical trainees, and all practised in settings with extracorporeal membrane oxygenation capabilities.

**Intervention:**

Respondents were asked to complete a web-based survey. Five of the survey questions were devoted to background demographic data, and seven questions were aimed at identifying physiologic targets. Two of the seven questions were in relation to a provided clinical vignette.

**Measurements and main results:**

This survey demonstrated that there is a lack of consensus in the management of patients after the bidirectional Glenn surgery. Specifically, granular SpO_2_, mean arterial pressure, and pH Goals were all less than 75% consensus. This survey highlights the variable practice patterns in providers taking care of patients after the bidirectional Glenn surgery, and further demonstrates the need for physiologic and outcome-driven targets to optimise the post-operative care.

## Introduction

The Bidirectional Glenn surgery has been shown to be an important staging procedure for patients born with single ventricle physiology. Approximately 1000 children are born each year in the United States with CHD.^1–3^ Mortality rates after the bidirectional Glenn surgery are relatively low at 2–4%.^4,5^ Despite the low mortality, the morbidity remains quite high and variable, with literature citing complication rates ranging from 20–44%.^6,7^ These morbidities are not only a burden to patients and families, but importantly, may make patients unfavourable candidates for further necessary cardiac interventions. Approximately 9–20% of patients are characterised as not being suitable candidates for additional cardiac interventions after the bidirectional Glenn surgery due to significant pre- and post-operative morbidity.^8–10^

Due to the variable mortality and high complication risks, authors have evaluated risk factors associated with unfavourable outcomes. Commonly cited anatomical and physiologic risk factors associated with increased morbidity and mortality include age at time of repair, pulmonary artery disease, atrioventricular regurgitation, ventricular function and ventricular morphology, prolonged initial cardiac repair, underlying medical or genetic conditions and prematurity.^11–20^ The goal of the bidirectional Glenn surgery is to provide stable pulmonary blood flow while decreasing the volume load on the single ventricle, allowing for greater oxygenation to the brain and other vital organs as the baby grows and develops. The bidirectional Glenn surgery is proven to be lifesaving in the care of children with SV physiology.^21,22^

All patients who undergo the bidirectional Glenn surgery recover in the Paediatric Cardiac Intensive Care Units and are extensively monitored and observed. Patients recovering after the bidirectional Glenn surgery have significantly deranged physiology, but the rigorous evaluation of physiologic targets linked to optimal outcomes has not been extensively studied.^23–26^ Even less is known about the practice patterns surrounding the management of these patients, and there is likely significant practice variation in the pre- and post-operative management of these patients.^5,27^

To understand the need for identifying optimal physiologic targets and establishing more granular post-operative practice guidelines for patients recovering after the bidirectional Glenn surgery, we sought to understand the current post-operative practice patterns through a survey of paediatric cardiac intensive care providers.

## Materials and methods

We conducted a national cross-sectional survey of physicians and health care providers to better understand practice patterns in the post-operative management of patients recovering after the bidirectional Glenn surgery.

We distributed our survey through an email distribution list of clinical paediatric cardiac intensive care staff through the Paediatric Cardiac Critical Care Consortium (PC^4^). This email list is comprised of paediatric cardiac intensive care physicians, advanced care providers, nurses, and training fellows across the United States, practising in various institutions. Contacts were encouraged to forward this survey to other care providers. We utilised survey methodology to develop, test, and administer our survey.^28,29^ Our survey was optional, with no identifying information obtained, to theoretically improve survey completion. No identifiable data was collected about each individual completing the survey or as to which institution they work. This study was approved with a waiver of informed consent by the University of Columbia Institutional Review Board (IRB: AAAV1905).

### Survey development

We developed questions to address clinicians’ practice patterns in the management of patients after the bidirectional Glenn. Our questions were specifically geared toward post-operative physiologic targets for optimal recovery after the bidirectional Glenn. Namely the questions focused on ventilation strategy, goal pH, goal oxygen saturation, and goal blood pressure. We provided a specific case scenario and asked follow-up questions to simulate further practice patterns.

There was a total of twelve questions; five questions were devoted to basic background demographic information of the individuals being sampled. The demographic data asked respondents to report their position, years of experience, the size of the paediatric cardiac intensive care units they work in, the presence of trainees (specifically fellows), and whether Extracorporeal Membrane Oxygenation is available. The seven clinical questions were specific to post-operative bidirectional Glenn management. Two of the seven clinical questions were in relation to a hypothetical case of a patient recovering post-operative day 3 from the bidirectional Glenn (see supplemental below).

In the clinical scenario (6-month-old with a history of Hypoplastic Left Heart Syndrome due to Mitral Stenosis and Aortic Atresia who initially underwent a Norwood-Sano now recovering after an uncomplicated bidirectional Glenn procedure), we queried respondents regarding their management intent in troubleshooting hypoxaemia, as well as target arterial partial pressure of carbon dioxide (paCO_2_).

### Survey testing

To test the survey’s goals and clarity, we administered the survey in person to six paediatric cardiac intensive care providers. The survey was modified after each phase of testing. The goal of the survey was to last no more than 10 minutes to maximise full completion per respondent.

### Survey formatting

The survey consisted of twelve questions. The survey included an introduction (stating the purpose), listed a contact person for subsequent questions, and then issued the questions. The practice management questions were presented first, followed by a clinical scenario with two follow-up questions. After respondents answered the clinical practice questions, five baseline demographic questions were prompted.

### Survey administration

The survey was distributed via email with a URL link to the Qualtrics platform (Qualtrics, LLC, Provo, UT). This platform allows de-identified tracking of respondents, to ensure that there is only one response per respondent. Participation was voluntary. Responses did not contain any identifiable information about the individual or where the individual practices. The responses were stored in a password-protected database on an encrypted server.

### Statistical analysis

Continuous variables are reported as mean, (Standard Deviation (SD)) and categorical variables as counts and percentages. We assumed that answers between correspondents were independent and that respondents did not discuss the questions amongst one another. Statistical analyses were performed using MATLAB 2020a (MathWorks, Massachusetts).

## Results

### Respondent characteristics

The survey was distributed to the email distribution list in October 2022. Two emails were sent, two weeks apart, encouraging individuals to complete the survey. There was a total of 39 responses; however, 4 individuals did not complete the entire survey, and their partial responses were removed, leaving a total of 35 completed surveys available for analysis. The average response time was 16.4 minutes. Twenty-eight (80%) of the respondents were paediatric critical care physician attendings, five (14%) were advanced practice providers (i.e. PAs, NPs), and two (6%) were clinical physician fellows.

On average, respondents had worked 7.86 years in paediatric critical care. All the respondents worked at institutions that offered extracorporeal membrane oxygenation. Thirty-two (91%) worked in institutions that had either paediatric cardiology or paediatric critical care clinical fellows. Twenty-one of the respondents (61%) practised in a paediatric cardiac intensive care setting with 21 or more beds, followed by six who worked in a unit with 16–20 beds (17%). There were four individuals (11%) who worked in Pediatric Intensive Care Unit (PICU) settings that contained 1–10 beds and 11–15 beds, respectively (Table 1).

**Table 1.** Demographic information of respondents

Baseline demographic data of individuals who responded to the practice survey				
**Role**	Attending	Fellow	APP	
*n*, (%)	28 (80)	2 (6)	5 (14)	
**PICU size**	21 + Beds	16–20 Beds	11–15 Beds	1–10 Beds
*n*, (%)	21 (60)	6 (17)	4 (11.4)	4 (11.4)
**Average years of training of all respondents**				
Years (IQR)	7.86 (8)			
**Trainees present**				
Yes : No	32:3			
**ECMO present**				
Yes : No	35:0			

### Practice patterns

Most of the respondents, twenty-one (60%), indicated that their post-operative bidirectional Glenn patients are extubated in the PICU, eleven (31%) reported that their patients are extubated in the OR, and three (9%) respondents indicated that there is a 50%/50% split of where patients are extubated (Table 2).

**Table 2.** Practice patterns of providers regarding the management of the post-operative bidirectional Glenn patient

Response to survey to assess practice patterns					
**Location of extubation**	Extubated in OR	Extubated in PCICU	50 / 50 PCICU vs. OR		
*n*, (%)	21 (60.0)	11 (31.4)	3 (8.6)		
**Goal SpO**_**2**_	70–75%	76–80%	81–85%	86–90%	Other
*n*, (%)	1 (2.9)	8 (22.9)	23 (65.7)	2 (5.7)	1 (2.9)
**Goal MAP**	MAP 41–50	MAP 51–60	MAP > 60	No specific goal	Other
*n*, (%)	2 (5.7)	25 (71.4)	1 (2.9)	4 (11.4)	3 (8.5)
**Goal pH**	7.26–7.30	7.31–7.35	>7.36		
*n*, (%)	4 (11.4)	15 (42.9)	16 (45.7)		
**Goal PEEP**	PEEP 5	PEEP 4			
*n*, (%)	34 (97.1)	1 (2.9)			
**Clinical question**	Management of a patient after the BDG with post-operative hypoxaemia				
**Choice**	Extubate	Diuresis	Catheterisation	Other	Medication
*n*, (%)	21 (77)	3 (8.6)	2 (5.7)	2 (5.7)	1 (2.9)
**Goal pCO2**	35–45	46–55	56–65		
*n*, (%)	6 (17.1)	24 (68.6)	4 (11.4)		

In the clinical case, for the patient recovering post-operatively with relatively hypoxaemia, twenty-seven (77%) respondents indicated that they would favour extubation, three (8.5%) respondents indicated that they would favour additional time for diuresis, two (5.7%) individuals would favour obtaining more information via a cardiac catheterisation, and two (5.7%) providers would favour other interventions (i.e. obtain additional imaging, optimise diuresis) and one respondent (2.8%) wanted to add additional medications.

**Figure 1.**
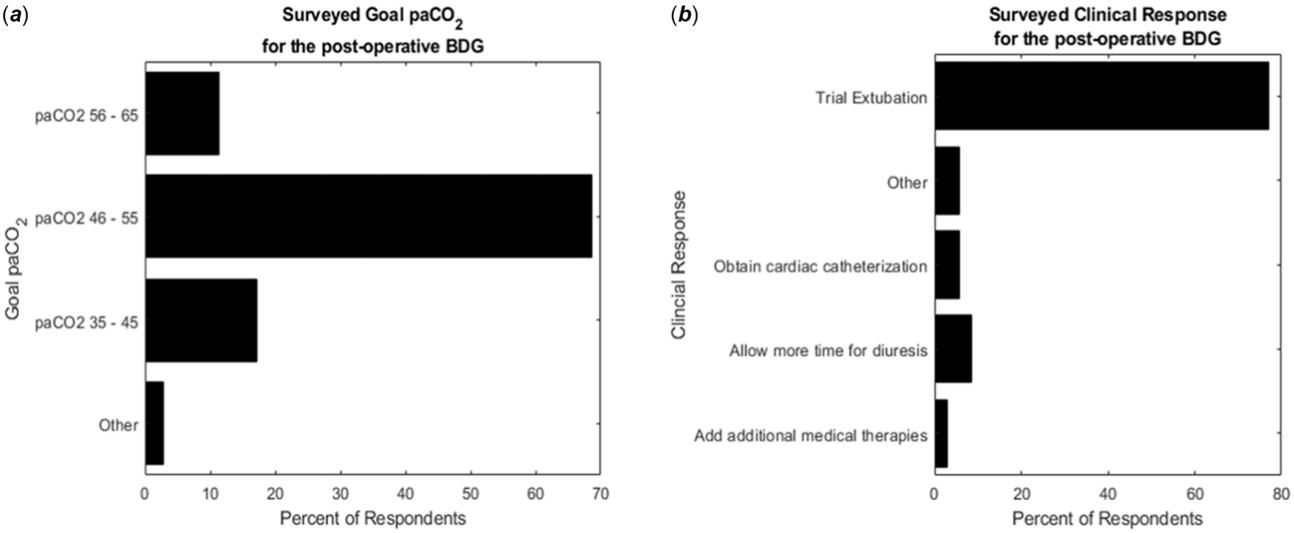
Percent of respondents who selected each (***a***) targeted pCO_2_ based on the clinical scenario for a hypoxemic patient who is intubated, post-operative day three, after the BDG surgery and (***b***) intervention based on the clinical scenario.

With the same clinical prompt, we investigated whether individuals have a target partial pressure of arterial carbon dioxide (paCO_2_) post-operatively in a hypoxemic patient after the bidirectional Glenn. Twenty-four (68.6%) had a goal paCO_2_ of 46–55 mmHg, six (17%) had a goal paCO_2_ of 35–45 mmHg, and four (11.4%) had a goal paCO_2_ of 56–65 mmHg. One person selected “Other” (2.9%) (Figure 1 **)**.

### Physiologic targets

**Figure 2.**
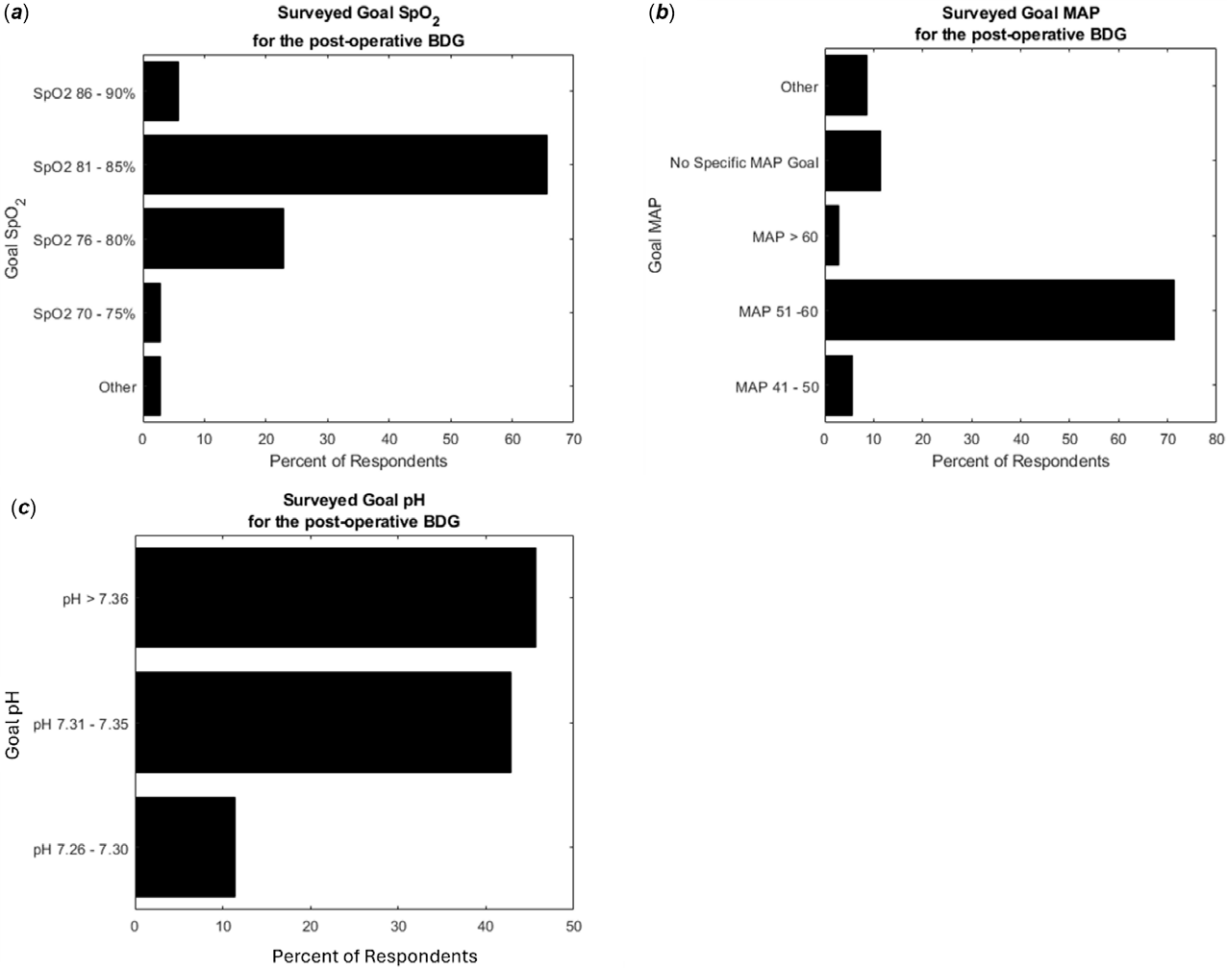
Post-operative goals set by the clinical providers in the management of the bidirectional Glenn patient. Graphs represent the percent of respondents who selected the respective management goal for (***a***) SpO2, (***b***) MAP, and (***c***) pH.

In the series of questions that surveyed post-operative physiologic targets after a patient with an uncomplicated bidirectional Glenn surgery, twenty-three (65.7%) respondents target a goal pulse-oximeter saturation of 81–85%, eight (22.9%) individuals target a pulse-oximeter saturation of 76–80%, two (5.7%) providers target a saturation of 86–90%, one (2.9%) respondent targeted a saturation of 70–75%, and one selected a broader range by selecting the “Other” option (75–85%). When questioned about a goal pH for the post-operative bidirectional Glenn patient, sixteen (45.7%) respondents indicated that they target a pH > 7.36, fifteen (42.9%) individuals target a pH ranging from 7.31 to 7.35, and four (11%) people target a pH ranging between 7.26 and 7.30. Immediately post-operatively, thirty-four (97%) respondents use a ventilator with Positive End Expiratory Pressure set to 5. Regarding blood pressure targets, twenty-five (71.4%) of the respondents target a Mean Arterial Pressure ranging from 51–60 mmHg, four (11.4%) providers indicated that they do not use a specific mean arterial pressure, three (8.6%) providers indicated that their specific mean arterial pressure is driven by other clinical settings, two (5.7%) individuals stated that their goal mean arterial pressure ranges from 41–50 mmHg, and one (2.9%) indicated that they target a mean arterial pressure > 60 (Table 2), (Figure 2).

## Discussion

This is the first study that attempts to characterise practice patterns and physiologic targets surrounding the care of patients recovering after the bidirectional Glenn surgery. Our study highlights both the common practices and the variability in management which occurs in the absence of rigorous data or studies.

The respondents were primarily paediatric cardiac intensive care attendings with greater than 5 years of experience. The majority of respondents have fellow trainees. We chose to include attendings, fellows, and advanced practice providers, to try and capture management patterns as robustly as possible. The respondents practice in an institution with greater than 21 bed units, indicating that these responses are from individuals who practice at academic institutions. Given the methodology for which we distributed this survey, it was impossible to determine how many individuals were contacted, and thus our response rate is unknown.

Despite the widely held belief that bidirectional Glenn patients have better cardiorespiratory haemodynamics with negative pressure breathing, this survey indicated that most patients are extubated in the paediatric cardiac intensive care units, rather than the OR. This is contrary to evolving literature that early extubation (i.e. <24 h) and/or extubation in the OR is associated with improved outcomes and shorter length of stay.^4,30–32^ It is important to note, however, that the decision to extubate a patient in the operating room is predicated on a multitude of variables, including length of the operation, use of cardiopulmonary bypass, technical difficulty, patient haemodynamics, as well as many other factors. The purpose of this study was not to try to elucidate which factors are impediments to early extubation, but rather to note the variable patterns observed when caring for this patient population.

The doctrine that children with bidirectional Glenn physiology have better outcomes with less positive pressure breathing was reflected in 97% of respondents reporting using a low positive end expiratory pressure of 5 for their intubated bidirectional Glenn patients. The notion of improved pulmonary blood flow, augmented by negative pressure breathing, was also emphasised by the clinical vignette, where 77.1% of respondents would favour extubating a relatively hypoxemic patient after their bidirectional Glenn surgery. Though the majority chose extubation, others chose to prolong intubation, obtain more imaging and or perform invasive procedures, demonstrating the often-inconsistent care these patients receive. These questions highlight how nuanced hypoxaemia in patients recovering after the bidirectional Glenn surgery can be. While the goal of these questions was to target traditional post-operative mechanical ventilation strategies; patient-specific variables (such as presence of veno-venous collaterals, pulmonary vein desaturation, decreased pulmonary blood flow, etc.) undoubtedly impact clinical decision making, and such complicated decisions do not translate perfectly to a relatively simple survey question.

None of the five responses regarding physiologic targets met consensus by Delphi methodology (i.e. >80% agreement).^33^ Some of the seminal work surrounding this patient population has demonstrated that cerebral blood flow and subsequent cavopulmonary anastomosis blood flow are improved with higher paCO_2_ values.^23,25,26,34,35^ This dogma was evident as most respondents (68.6%) indicated that they target a slightly higher paCO_2_. Understanding the ideal paCO_2_ for patients recovering after the bidirectional Glenn operation could have key implications. Precise management of paCO_2_ may improve neurologic outcomes, as CO2 has a profound effect on cerebral blood flow and cerebral autoregulation.^23,36,37^

Similarly, given the notion of permissive hypercarbia to promote cerebral blood flow, there is an accepted doctrine that permissive acidemia is also beneficial in the management of the post-operative bidirectional Glenn patient. The respondents were nearly split as to target a normal pH (45.7%) or a slightly acidic pH (42.8%). It could be argued that a pH range of 0.05 (i.e. 7.26 to 7.30) may be too strict in real clinical practice. In secondary analysis, we dichotomised survey responses to either a goal pH range of 7.20 to 7.30 or to a goal pH range of greater than 7.31. Using this methodology, 14% (5 of 35) providers chose a pH ranging from 7.20 to 7.30, while 86% (30 of 35) providers chose a pH greater than 7.31. In the presence of limited studies and limited recent studies containing granular physiologic studies, the intensivist is tasked with choosing the best treatment goals based on their patient’s current physiology. This study shows that even with these limited studies, there are heterogeneous practice patterns, while most providers target a pH greater than 7.31.

There is a paucity of guiding literature surrounding goal oxygenated haemoglobin saturation and blood pressure targets in the post-operative period. At the bedside, the care team constantly evaluates and monitors the post-operative patient. Goals are set relatively haphazardly, as is seen when respondents were asked to choose their goal for mean arterial pressure. Seventy-one per cent of respondents chose a mean arterial pressure of 51–60; there was a wide range of goal mean arterial pressures amongst the remainder, highlighting variable practice patterns.

The hallmark of the bidirectional Glenn surgery is to provide more stable pulmonary blood flow for the growing child. The amount of blood flow and the degree of improvement in haemoglobin oxygen saturation, especially in the immediate post-operative period, have not been rigorously explored. This is made evident by our question, where 65.7% of respondents chose a goal haemoglobin oxygen saturation (SpO_2_) of 81–85%, but the remaining responses spanned the range of haemoglobin oxygen saturation levels from 70 to 90%. In clinical practice, a 5% SpO_2_ range may be too restrictive and impractical. On secondary analysis, we binned responses into 10% SpO_2_ ranges. We found that 20% of providers (7 of 35) target a SpO_2_ between 70 and 80%, and 80% of providers (28 of 35) target a SpO_2_ between 80 and 90%.

This is the first study that attempts to identify practice patterns of clinical providers in the post-operative management of a patient recovering after the bidirectional Glenn. Respondents were identified through an email distribution list composed of paediatric cardiac intensive care providers; thus, the responses and opinions are likely to be valid. Despite these strengths, our survey did not have a robust number of respondents. Based on our survey distribution methodology, it is impossible to deduce the response rate. The limited number of respondents is not overall surprising given the complexity and nuances of this patient population. Therefore, the responses may not be truly indicative of the nationwide practice patterns. In an aim for anonymity, we did not ask individuals to report which institution they work in; thus, it is possible that our selection represents several institutions rather than a more diverse population. By including attendings, fellows, and advanced practice providers, we hoped to capture practice patterns as broadly as possible. Furthermore, stated practices may not actually mirror the decisions that happen in real time at the bedside. This point is mitigated by the fact that we asked respondents to identify their personal goals and beliefs rather than what may be the institution’s. Lastly, the anatomy of patients recovering after the bidirectional Glenn operation can encompass a wide range of physiology. We assumed that respondents deduced that we were surveying practice patterns regarding patients with an isolated superior cavopulmonary anastomosis, without antegrade pulmonary blood flow (i.e. pulsatile Glenn, or “Super Glenn”). Not being explicitly clear may have impacted some individual’s responses. Irrespective of the limitations, this survey demonstrates heterogeneous responses to basic principles in the care of this complex cardiac population, warranting further investigation into studies to identify optimal physiologic targets for improving the care and outcomes we provide to this at-risk population.

## Conclusion

Although patients routinely do well after their bidirectional Glenn surgery, our survey highlights the variable practice patterns surrounding the management of these patients. Given the plethora of data available for the intensivist at the bedside, clear goals and metrics could be created to better inform and guide the treatment team at the bedside. Leveraging the data available may lead to more standardised care for these complex patients, with an aim to improve their overall cognitive and functional long-term outcomes.

## Supporting information

10.1017/S104795112511113X.sm001 Weinerman et al. supplementary material Weinerman et al. supplementary material

## Supplementary material

The supplementary material for this article can be found at https://doi.org/10.1017/S104795112511113X
