# Supplementary material for: Post-operative practice patterns after the bidirectional Glenn surgery: a survey
Source: Cardiol Young. 2026 Jan 23:1–7. Online ahead of print. doi: 10.1017/S104795112511113X (PMC12885051; doi:10.1017/S104795112511113X)
Supplement: Weinerman et al. supplementary material [file S104795112511113Xsup001.docx]

**Supplemental Material**

**Post-operative practice patterns in managing a patient after the Bidirectional Glenn Operation: A Survey**

Thank you for taking part in this anonymous, brief survey. In total this survey should take you 5-10 minutes. I am a pediatric critical care doctor at Morgan Stanley Children's Hospital - Columbia University doing research in single ventricle patients. This survey's aim is to gain insight into practice patterns in post-operative management of bidirectional Glenn (BDG) patients across various institutions.

Your answers are anonymous and will not be linked to any identifiable data. If you have any questions or concerns, please do not hesitate to contact me, Bennett Weinerman ([bw2681@cumc.columbia.edu](mailto:bw2681@cumc.columbia.edu)). Please forward this to any and all physicians who care for post-operative bidirectional Glenn’s at your institution. I greatly appreciate your participation.

For the majority of your post-operative **bidirectional Glenn** patients, are they extubated in the operating room (OR), or are they extubated in the cardiac ICU?

- Extubated in the OR
- Extubated in cardiac ICU
- 50% extubated in the OR, 50% extubated in the ICU

Immediately post-operatively, (i.e. post-op day 0 to post-op day 1), what are your **goal pulse oximeter saturations (SpO_2_)** for an **uncomplicated 6-month-old bidirectional Glenn** patient with no atrioventricular valvular regurgitation, good function, and unobstructed Glenn flow?

- SpO_2_70 – 75
- SpO_2_76 – 80
- SpO_2_ 81 – 85
- SpO_2_ 86– 90
- SpO_2_ >91
- Other

Immediately post-operatively, (i.e. post-op day 0 to post-op day 1), what is your **goal pH** for an **uncomplicated 6-month-old bidirectional Glenn** patient with no atrioventricular valvular regurgitation, good function, and unobstructed Glenn flow?

- pH 7.20 – 7.25
- pH 7.26 – 7.30
- pH 7.31 – 7.35
- pH > 7.36
- Other

A **Bidirectional Glenn** is intubated for relative hypoxemia.

- They are inhaled nitric oxide
- Cardiac function is optimized with vaso-active support and diuretics
- Chest xray looks well expanded

What is the typical Positive End Expiratory Pressure (PEEP), that you use?

- PEEP 0
- PEEP 2
- PEEP 3
- PEEP 4
- PEEP 5
- Other

Immediately post-operatively, (i.e. post-op day 0 to post-op day 1), what is your **goal Mean Arterial Pressure** for an **uncomplicated 6-month-old bidirectional Glenn** patient with no atrioventricular valvular regurgitation, good function, and unobstructed Glenn flow?

- MAP 30 – 40
- MAP 41 – 50
- MAP 51 – 60
- MAP > 60
- No Specific MAP Goal
- Other

A 6 month old male with HLHS (Mitral Stenosis, Aortic Atresia) who initially underwent a Norwood-Sano, is recovering after his uncomplicated bidirectional Glenn procedure now POD#3.

- pre-Glenn cath: patent pulmonary arteries, normal function and normal PVR (pulmonary vascular resistance).
- post-operative TEE:  moderate AVV -Regurgitation, normal function and unobstructed Glenn flow.
- Chest Xray (POD3): No pleural effusion or pneumothorax
- TTE (POD3): Unchanged

He is intubated for relative hypoxemia. His ventilator has been optimized. He has had adequate diuresis. 

SpO2: 74% on inhaled nitric oxide
Otherwise vitals and hemodynamics normal for age
Medication: milrinone infusion
Labs: Hemoglobin 12, hematocrit 40

He has the following arterial blood gas

| pH | 7.33 |
| --- | --- |
| paCO2 | 50 |
| paO2 | 50 |
| HCO3 | 25 |
| Base Excess | -1.00 |
| Lactate | 0.50 |

What would be your next step in management?

- Obtain Cardiac Catheterization
- Trial Extubation with either noninvasive pressure ventilation or supplemental oxygen
- Add additional medical therapies
- Allow more time for diuresis
- Open the chest to allow for improved lung compliance
- Other

In the same clinical scenario as above, what are your target carbon dioxide (paCO_2_) levels for a hypoxemic (~74%) intubated bidirectional Glenn patient?

- paCO_2_ 35 – 45
- paCO_2_ 46 – 55
- paCO_2_ 56 – 65
- paCO_2_ > 65
- Other

How many beds is your Pediatric Cardiac Care Unit?

- 1 – 10 Bed Unit
- 11 – 15 Bed Unit
- 16 – 20 Bed Unit
- 21+ Bed Unit

What is your current position?

- Attending
- Fellow
- Resident
- Other

How long have you been in your current position?

- Whole integer entered

Does your institution offer ECMO (Extracoporeal Membrane Oxygenation)?

- Yes
- No

Does your cardiac ICU have ICU and/or Cardiology fellows in it?

- Yes
- No
